# Heterogeneity in major depression and its melancholic and atypical specifiers: a secondary analysis of STAR*D

**DOI:** 10.1186/s12888-021-03444-3

**Published:** 2021-09-16

**Authors:** Lorenzo Lorenzo-Luaces, John F. Buss, Eiko I. Fried

**Affiliations:** 1 Department of Psychological and Brain Sciences, Indiana University, Bloomington, 47405 IN USA; 2 Department of Psychology, Leiden University, Leiden, 2333 AK Netherlands

**Keywords:** Depression, Classification, Melancholia, Atypical

## Abstract

**Objectives:**

The melancholic and atypical specifiers for a major depressive episode (MDE) are supposed to reduce heterogeneity in symptom presentation by requiring additional, specific features. Fried et al. (2020) recently showed that the melancholic specifier may increase the potential heterogeneity in presenting symptoms. In a large sample of outpatients with depression, our objective was to explore whether the melancholic and atypical specifiers reduced observed heterogeneity in symptoms.

**Methods:**

We used baseline data from the Inventory of Depression Symptoms (IDS), which was available for 3,717 patients, from the Sequenced Alternatives to Relieve Depression (STAR*D) trial. A subsample met criteria for MDE on the IDS (“IDS-MDE”; N =2,496). For patients with IDS-MDE, we differentiated between those with melancholic, non-melancholic, non-melancholic, atypical, and non-atypical depression. We quantified the observed heterogeneity between groups by counting the number of unique symptom combinations pertaining to their given diagnostic group (e.g., counting the melancholic symptoms for melancholic and non-melancholic groups), as well as the profiles of DSM-MDE symptoms (i.e., ignoring the specifier symptoms).

**Results:**

When considering the specifier and depressive symptoms, there was more observed heterogeneity within the melancholic and atypical subgroups than in the IDS-MDE sample (i.e., ignoring the specifier subgroups). The differences in number of profiles between the melancholic and non-melancholic groups were not statistically significant, irrespective of whether focusing on the specifier symptoms or only the DSM-MDE symptoms. The differences between the atypical and non-atypical subgroups were smaller than what would be expected by chance. We found no evidence that the specifier groups reduce heterogeneity, as can be quantified by unique symptom profiles. Most symptom profiles, even in the specifier subgroups, had five or fewer individuals.

**Conclusion:**

We found no evidence that the atypical and melancholic specifiers create more symptomatically homogeneous groups. Indeed, the melancholic and atypical specifiers introduce heterogeneity by adding symptoms to the DSM diagnosis of MDE.

## Background

Experiences of depressed mood or low positive affect can range from states of transient sadness to highly debilitating, chronic, and recurrent patterns of symptoms [1, 2]. According to the Diagnostic and Statistical Manual of Mental Disorders (DSM), a major depressive episode (MDE) requires a minimum of five symptoms, one of which must be depressed mood or anhedonia (i.e., loss of interest or pleasure) for at least two weeks [3]. Most MDE symptoms are compound criteria that vary qualitatively (e.g., diminished ability to think, or to concentrate, and indecisiveness are all counted as the same symptom”) or consist of complaints in the opposite direction (e.g., sleep disturbances manifesting as either insomnia or hypersomnia).

The optimal classification of major depressive disorder (MDD), the diagnosis most commonly associated with a MDE [4], has been one of the major challenges in the history of psychiatry [1, 5–14]. The DSM diagnostic criteria for a MDE are a polythetic set (i.e. there are more symptoms than necessary for a diagnosis). Thus, there can be considerable heterogeneity in the symptom presentation of MDD to the point that two individuals with the diagnosis may not overlap on any one symptom [15, 16]. Counting compound criteria as a single symptom, there are 227 possible ways of meeting criteria for a MDE [15, 16]. In a sample of 1,566 psychiatric outpatients, Zimmerman et al. [16] reported that 170 of the 227 profiles were represented. Being more liberal, and perhaps more accurate, in counting the compound symptoms as distinct symptoms, there are as many as 10,377 ways of meeting the MDE criteria [17]. In a sample of 3,703 outpatients, Fried & Nesse [15] had symptom data that allowed for up to 4,096 possible profiles of the symptoms. Of these, 1,030 were identified in the data (25.1%). Underscoring the importance of attending to heterogeneity in symptoms are findings that symptoms have different relations to validators like impairment [18] co-morbidity and temperamental vulnerabilities [5, 19] as well as to biological vulnerabilities [20].

The DSM provides the option to identify “more homogeneous” (p. 21) subgroups of patients via subtypes and specifiers [3]. Subtypes are defined as mutually exclusive categories like “predominantly hyperactive/impulsive” attention-deficit and hyperactivity disorder (ADHD) vs. “predominantly inattentive” ADHD. Specifiers are not mutually exclusive, such as seasonal-affective and atypical depression. For the diagnosis of a MDE, the DSM differentiates between diagnostic categories (i.e., bipolar vs. unipolar), illness history (i.e., recurrent vs. single episode), and symptom severity (i.e., mild, moderate, and severe) while containing nine different specifiers for course or symptom presentation (e.g., catatonia, anxious distress). Of these specifiers, melancholia and atypical depression features are among the oldest and most widely studied [21], and are the focus of the present paper.

Melancholia is characterized primarily by a loss of positive affectivity, manifested either in the loss of pleasure in almost all activities or a lack of mood improvement in the context of positive events. The melancholic specifier was first formally operationalized in DSM-III [22] but was meant to capture the historical conceptualizing depression, which differentiated between milder forms of depression, usually assumed to be psychogenic or triggered by a negative event, and depression without an apparent cause [8, 9, 23]. Melancholia is commonly cited in the literature as being a sub-classification of depression whose onset and maintenance has a greater contribution from biological vulnerabilities [6].

Atypical depression is characterized, in juxtaposition to melancholia, by the ability to experience mood improvements as well as a longstanding pattern of interpersonal sensitivity. The “atypical features” specifier was formally introduced in DSM-IV [24], but comports to prior publications which identified a subgroup of patients who may have a specific response to treatments [25], though this pattern of results has not been replicated [26, 27]. Some evidence supports the idea that atypical features may be related to psychosocial vulnerabilities, like early adversity and neuroticism [5, 19], as well as to biological vulnerabilities, including the presence of metabolic syndrome [28]. More detailed information on the history, validity, and debates surrounding melancholia and atypical depression can be found elsewhere [27, 29–34].

Fried et al. [17] recently challenged the assumption that specifiers identify more homogeneous subgroups of patients. Following this work, we computed the total number of possible symptom profiles for MDD vs. MDD plus the melancholic specifier. As stated, the number of possible symptom profiles for meeting a MDE criteria were either 227 to 10,377, depending on how “compound” symptoms were treated (e.g., whether psychomotor agitation and retardation are conceptualized as two different symptoms or just one manifestation of motor disturbances). However, the total number of symptom profiles for MDD plus melancholia ranged from 10,999 to 341,737. These calculations demonstrate that there are more potential ways to meet for the melancholic specifier than a MDE alone, which contrasts with the DSM’s explicit goal of identifying more homogeneous group of patients. If the DSM specifiers do not achieve their intended purpose of creating more homogeneous subgroups, it is possible that they may not help elucidate biopsychosocial mechanisms underlying different forms of depression.

### Study objectives

The theoretical analyses of Fried et al. [17] suggest there are more possible ways to meet diagnostic criteria for melancholia than for MDE. However, to our knowledge, no empirical research has quantified whether in practice (i.e., in presenting symptoms in outpatient samples), the MDE specifiers melancholic and atypical depression reduce observed heterogeneity. Our objective was to explore whether the atypical and melancholic subtypes reduced observed symptom heterogeneity, using data from a large sample of outpatients. We followed the procedures used in prior work, which involve counting the number of unique profiles of symptoms endorsed by patients. We refer to this unique combination of symptoms as “profiles.”

## Methods

### STAR*D

We reanalyzed the public-access dataset from the NIH-supported Sequenced Treatment Alternatives to Relieve Depression (STAR*D) study [35], which we downloaded from the National Institute of Mental Health Data Archive on September 16, 2019. STAR*D was a multi-site clinical trial conducted in the USA and designed to have greater external validity than treatment trials usually do [36, 37]. STAR*D treatment was designed as a stepped care protocol wherein patients received additional, usually more intensive, treatments if their symptoms had not improved at a prior level. In the first level stage, 4,041 patients were enrolled, and all participants received the selective serotonin reuptake inhibitor (SSRI) citalopram. Data were collected via telephone interviews. STAR*D was approved by the institutional review boards (IRBs) of all participating institutions, and after complete description of the study to the subjects, written informed consent was obtained. Prior STAR*D publications report on a subset of the 4,041 patients; excluding those who had mild symptoms or who did not provide data beyond the initial assessment [38]. Because our research question does not involve change in symptoms, and because we want to maximize the representativeness of our sample (i.e., to include those with mild symptoms) we did not exclude any patients from our analysis a priori.

### Participants

Inclusion criteria for STAR*D participants were: being between the ages of 18 and 75 years, meeting DSM-IV criteria for unipolar, non-psychotic MDD. MDD status was assessed by a checklist based on DSM-IV criteria [35, 39], after patients expressed interest in treatment for depression. Exclusion criteria were a history of mania or hypomania, schizophrenia, schizoaffective disorder, or psychosis, or current anorexia, bulimia, or primary obsessive-compulsive disorder (OCD), which were assessed with The Psychiatric Diagnostic Screening Questionnaire [40] via clinical interview. Further exclusion criteria and details about the study design are described elsewhere [35, 39]. Patients in STAR*D were excluded from some publications if they had scores ≤14 on the Hamilton Rating Scale for Depression (HRSD) [38], though these patients were assessed for baseline and their data was available at subsequent steps [35]. We analyze data for all STAR*D participants who had available IDS scores, even if they entered the trial with milder symptoms.

### Outcome measures

#### Inventory of depressive symptomatology (IDS)

We analyzed baseline data on the clinician-rated version of the IDS [41]. The IDS encompasses 30 depression symptoms, both DSM and non-DSM symptoms, rated on a 4-point (0-3) scale with a higher score indicating greater severity. Consistent with prior work [42], we considered a symptom to be present when an individual endorsed a severity level ≥2. The IDS covers most DSM-5 criterion symptoms in disaggregated form. For example, it queries both psychomotor agitation and psychomotor retardation. Nonetheless, we had to make several decisions regarding which variables to include in our analyses, discussed below.

#### Appetite or weight disturbances

Disaggregated information were not available for the two symptom domains “weight problems” and “appetite problems.” Instead, a patient was deemed to have either increased or decreased appetite or weight but could not rate both increases and decreases. We combined the responses to the appetite and weight questions, using the highest rating on either question to create two variables: appetite/weight decrease or appetite/weight increase. For example, if a participant was judged to have experienced weight loss, that individual was rating as having appetite/weight decrease but not appetite/weight increase. We coded the variables this way to avoid adding unnecessary heterogeneity (e.g., so two individuals who had severe appetite loss were not deemed as having a different symptom profile if one participant lost weight but the other did not).

#### Sleep disturbances

The IDS queries early, middle, and late insomnia as well as hypersomnia. We generally distinguished hypersomnia from insomnia. Distinguishing early insomnia from middle and late insomnia is necessary for the diagnosis of melancholic features. Nonetheless, to avoid inflating the degree of heterogeneity present in the symptom data, we consider all these examples of insomnia as a single symptom, as done in prior work [15], assigning each patient the higher-rated symptom they endorsed (i.e., if the highest symptom was ≥2 the patient was considered to have insomnia). We separately explored the presence of the early insomnia vs. other symptoms of insomnia only when counting the number of symptom profiles that include melancholic symptoms.

### Analytic strategy

All data were analyzed using the R programming language (code available at: https://osf.io/v8sbe/). From the 4,041 participants originally enrolled into STAR*D, 3744 (92.65%) patients provided early data during the first measurement point of the first treatment stage. We had full symptom-level IDS data on 3,717 patients, who represented 91.98% of all patients. Our aim was to count the number of symptom profiles across MDD and its melancholic and atypical specifier groups.

First, we present basic descriptive data on the categorical endorsement of all the symptoms we are studying, which include the symptoms from the DSM MDE criteria, the symptoms from the melancholic specifier, and the symptoms from the atypical specifier. We identify a subsample of patients who met criteria for MDD (2,496, 61.76%) using the IDS. This is lower than the number of cases with MDD in other STAR*D reports because we rely on the IDS rather than the STAR*D-specific checklist [35].

**Fig. 1:**
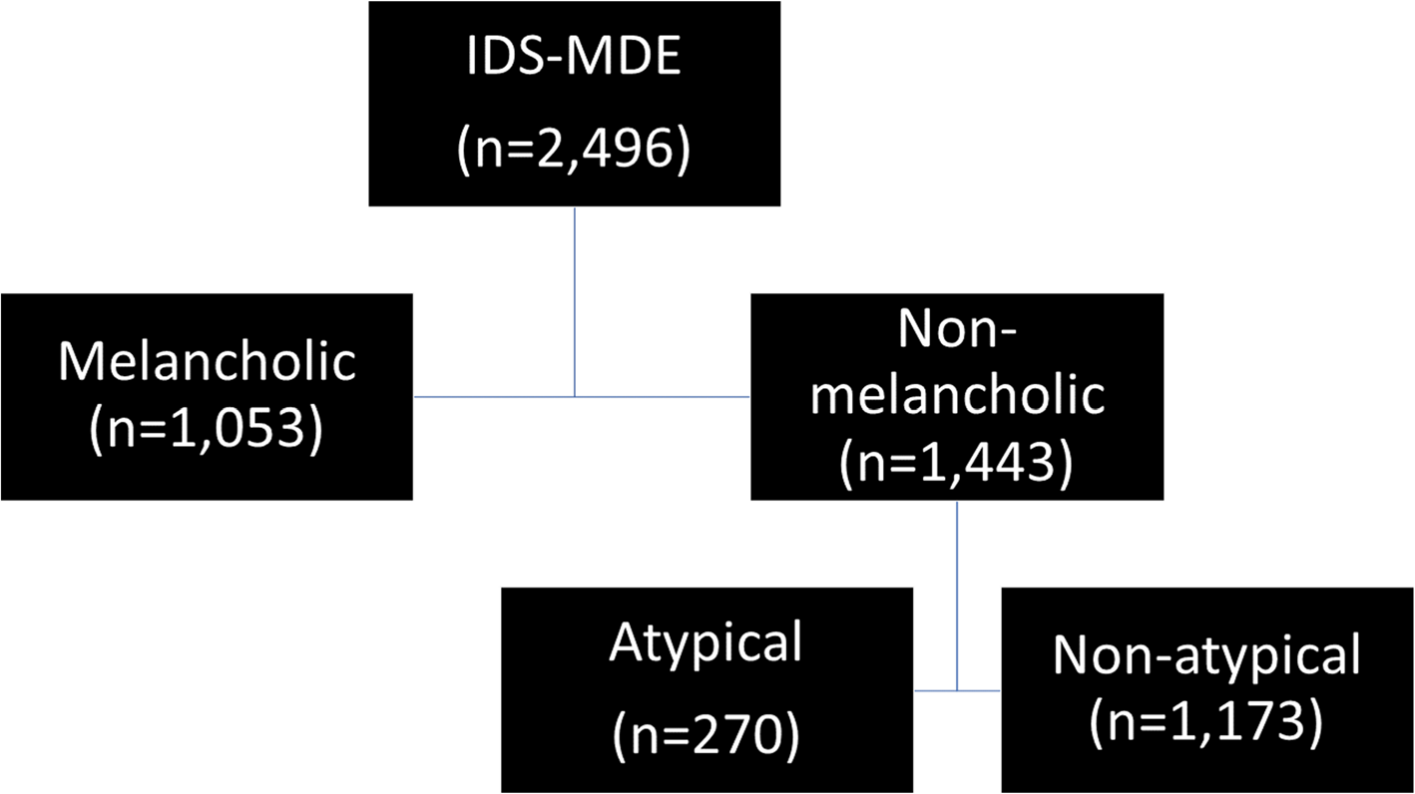
Star*D Participant Flowchart

To emulate the ways in which the DSM uses the specifiers, we created five groups (see Fig. 1). The IDS-MDE group consisted of patients who endorsed either sadness, loss of interest, or loss of pleasure, and a total of five DSM MDE symptoms. The second group, melancholic, featured patient who met IDS-MDE criteria plus who endorsed the melancholic specifier criteria. The melancholic criteria require the presence of either loss of pleasure or loss of mood reactivity, along with three symptoms from a list that includes: distinct quality of mood, depression that is worse in the morning, early-morning awakenings, psychomotor agitation or retardation, anorexia or weight loss, and excessive or inappropriate guilt. The third group was of patients who met IDS-MDE criteria but did *not* meet the melancholic specifier criteria (“non-melancholic”). The fourth group was of patients who met IDS-MDE criteria and met criteria for the atypical specifier. The atypical specifier requires the presence of mood reactivity along with two other symptoms from a list of four: weight gain or increase in appetite, hypersomnia, heavy or leaden feelings in the extremities, and a pattern of interpersonal rejection sensitivity outside the context of mood episodes. We respected the DSM’s hierarchical rules wherein a person cannot meet criteria for the atypical specifier if they meet criteria for the melancholic specifier. The final group was patients with non-melancholic, non-atypical depression (“non-atypical”).

For these 5 groups, we counted the total number of symptom profiles that created the groups, using the *distinct* command in the *dplyr* package. For the IDS-MDE group, we counted the total number of profiles of depressive symptom profiles using the DSM-MDE symptom criteria. For the melancholic and non-melancholic groups, we counted the total number of profiles of melancholic and depressive symptoms. For the atypical and non-atypical groups, we counted the total number of profiles using atypical and depressive symptoms.

To identify whether the differences in the number of profiles between the groups are statistically significant, we conducted permutation tests comparing the number of profiles in the specifier groups and their counterparts (i.e., melancholic vs. non-melancholic and atypical and non-atypical) across 10,000 permutations. In a permutation test, the variable of interest, here the group label (e.g., melancholic vs. non-melancholic), is randomized or permuted 10,000 times and the number of symptom profiles are counted in each of the permutation samples. Because the subgroup labels are assigned randomly in the permutations, this creates a distribution of symptom counts wherein there is no relationship between the label and the symptom counts. Any difference in the number of profiles that emerges between the melancholic vs. non-melancholic or atypical vs. non-atypical groups should be spurious (e.g., the product of sample size) and the p-value is the probability that the permutations contains values as extreme or more extreme than the observed number of profiles (e.g., if *p* = 0.03, then 300 out of 10,000 of the permutations produced as extreme a difference in the number of profiles). We tested the hypothesis that the differences in the number of profiles between the specifier and “non-specifier” groups (e.g., melancholic vs. non-melancholic) was different than would be expected by chance. Although the DSM suggests the melancholic and atypical groups should be less heterogeneous than their counterparts, our previous findings [17] suggest the opposite. Accordingly, in this test, we employed a two-tailed test with a *p* value of <0.05.

To quantify the magnitude of the differences while equating the groups on sample size, we randomly subsampled 100 patients from each of the five subgroups 5,000 times. This procedure provides a mean number of profiles per 100 patients for each of the diagnostic subgroups, along with a distribution of the mean number of profiles. If the groups are fully homogeneous, there should be 1 profile for every 100 patients. In a maximally heterogeneous group, there should be 100 profiles for every 100 patients.

By definition, the specifier groups differ in the number of symptoms that make up each group. Thus, in addition to counting the number of symptom profiles of DSM-MDE symptoms plus the specifier symptoms, we repeated the analyses above using only the DSM-MDE symptoms (e.g., sadness, adhedonia, difficulty concentrating, etc). Because in these analyses, the same number of symptoms are being considered for each group, any differences that emerge cannot be attributed to the number of symptoms. This was done for the IDS-MDE, melancholic, non-melancholic, atypical, and non-atypical groups.

Additionally, we conducted two sensitivity analyses. The DSM imposes a hierarchy on the diagnosis of the melancholic and atypical specifier wherein an individual cannot be diagnosed with the atypical specifier if they meet criteria for the melancholic specifier. Because this may bias results, we first repeated the analyses above by relaxing the DSM’s hierarchical rule. Second, we explored whether any individual symptom was associated with reduced heterogeneity as indexed by the ratio of unique profiles the symptom appeared in to the number of patient endorsements.

## Results

Table 1 shows the descriptive statistics representing endorsement of IDS symptoms as binary with the presence (≥2) or absence (0 – 1) of symptoms in the patients who had full IDS data and met IDS-MDE criteria (N =2,496). We focus our analyses on these patients. As seen in Table 1, sad mood (93.43%) and insomnia (91.11%) were the most frequently reported symptoms. The least frequently reported symptoms were psychomotor retardation (9.25%) and hypersomnia (14.82%). Of the patients with an IDS-MDE, 1,053 met criteria for melancholia (42.19%), and 270 met criteria for the atypical specifier (10.82%).

**Table 1:** Endorsement of specific symptoms of DSM criteria for major depression, melancholia, and atypical specifiers in patients with MDD, MDD with melancholic features, and MDD with atypical features, as determined by the IDS in STAR*D (N=2496)

	IDS-MDE		Melancholic		Atypical	
Symptom	%	(n)	%	(n)	%	(n)
Sad mood	93.43	2332	94.21	992	90.00	243
Lost interest	79.41	1982	82.34	867	70.00	189
Lost pleasure^a^	60.34	1506	79.68	839	35.56	96
Appetite/weight decrease^a^	45.15	1127	64.20	676	21.11	57
Appetite/weight increase^b^	27.24	680	19.66	207	57.78	156
Insomnia	91.11	2274	95.44	1005	86.67	234
Hypersomnia^b^	14.82	370	11.49	121	32.96	89
Psychomotor retardation^a^	9.26	231	14.82	156	6.67	18
Psychomotor agitation^a^	30.45	760	47.20	497	20.37	55
Fatigue	80.50	2009	79.68	839	82.96	224
Worthlessness/guilt^a^	76.60	1912	86.71	913	75.56	204
Diminished concentration	87.98	2196	88.70	934	87.04	235
Suicidality	19.79	494	25.55	269	12.59	34
Unreactive mood	57.53	1436	76.73	808	0.00	0
Distinct quality of mood	49.68	1240	64.77	682	47.78	129
Depression worse AM^c^	10.86	271	17.28	182	6.30	17
Early morning awakening	49.80	1243	72.27	761	34.07	92
Mood reactivity	42.47	1060	23.27	245	100.00	270
Leaden paralysis	32.29	806	33.24	350	59.26	160
Rejection sensitivity	51.88	1295	57.45	605	78.52	212

^a^also a symptom of the ‘melancholic features’ specifier,

^b^also a symptom of the ‘atypical features’ specifier,

^c^AM = in the morning

### Heterogeneity added by specifier symptoms

**Fig. 2:**
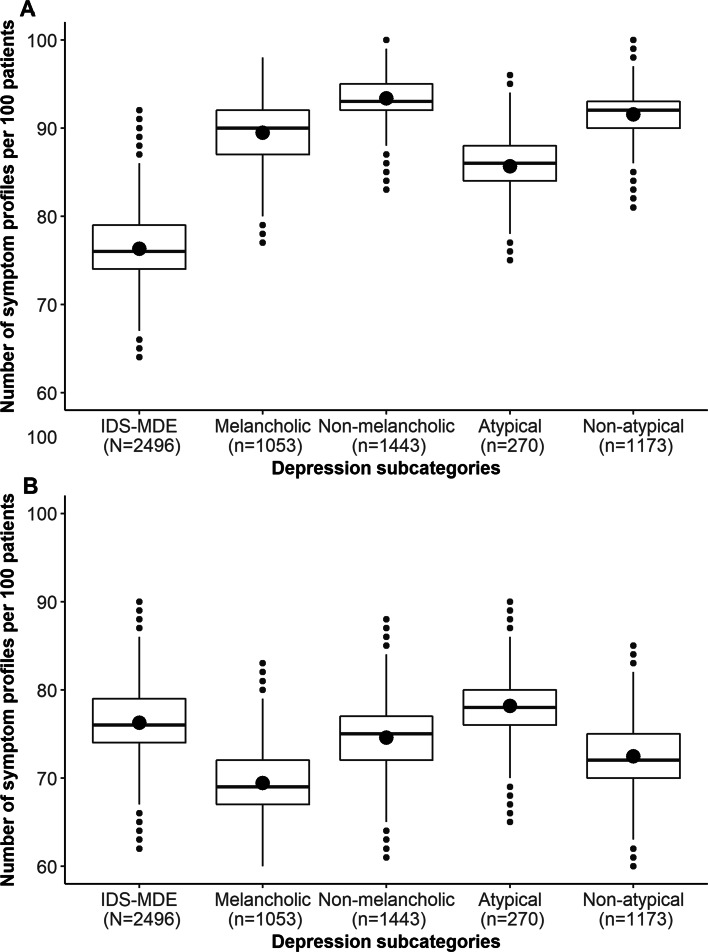
Number of unique symptom profiles of depression and its specifiers (Panel **A**) or depressive symptoms alone (Panel **B**) across 1,000 subsamples of n=100 for IDS-MDD, melancholic, non-melancholic, and non-atypical

When examining the observed number of symptom profiles, the atypical and melancholic specifier groups at first appeared to report fewer profiles than their non-atypical and non-melancholic counterparts. Specifically, the melancholic group (n =1,053) reported a total of 646 unique profiles of depression plus the melancholic specifier symptoms while the non-melancholic (n =1,443) reported 891 such profiles. These seeming differences are likely a product of sample size. First, the ratio of profiles to patients was comparable in the melancholic group (0.61) and non-melancholic group (0.62). Second, in a permutation test, the difference in the number of symptom profiles between the melancholic and non-melancholic subgroups was not statistically significant (*p* = 0.35). Finally, equating the groups on sample size by subsampling 100 patients multiple times (see Fig. 2A) suggested that not only were the differences not statistically significant but they were not clinically meaningful. Most of the melancholic (95.05%) and non-melancholic (96.86%) profiles were endorsed by five or fewer patients.

The atypical group (n =270) reported a total of 198 unique profiles of DSM-MDE symptoms plus the atypical specifier symptoms while the non-atypical group (n =1,173) reported 682 such profiles. Thus, the ratio of profiles to patients was somewhat *higher* in the atypical group (0.73) than in the non-atypical group (0.58, i.e., the non-atypical group appeared more homogeneous). In a permutation test, the difference in the number of profiles between the atypical and non-atypical subgroups was smaller than would be expected by chance (*p* <0.001; the group membership reduces heterogeneity less than would be expected by chance). Equating the groups on sample size by subsampling 100 patients multiple times (see Fig. 2A) suggested that these differences were not clinically meaningful. Most of the atypical (98.49%) and non-atypical (95.45%) profiles were endorsed by five or fewer patients

### Heterogeneity in depression symptoms alone

In the previous analyses, we investigated how specifiers could influence the number of unique symptom profiles reported by patients, though the specifiers differ in the number of symptoms they include and may thus be associated with different levels of heterogeneity as quantified by symptom count. To address this, we computed the total number of profiles of the symptoms of MDE in the IDS-MDD group, melancholic, non-melancholic, atypical, and non-atypical. As before, when focusing only on the symptom profiles, the atypical and melancholic specifier groups appeared to have fewer combination than their non-atypical and non-melancholic counterparts. Specifically, the melancholic group (n =1053) reported a total of 361 unique profiles of depressive symptoms while the non-melancholic (n =1,443) reported 453 such profiles. The ratio of profiles to patients was comparable in the melancholic group (0.34) and non-melancholic group (0.31). A permutation test showed that the difference in the number of symptom profiles between the melancholic and non-melancholic subgroups was not statistically significant (*p* = 0.64); and equating the groups on sample size (see Fig. 2B) suggested that results were neither statistically nor clinically meaningful. Most of the DSM-MDE profiles in the melancholic (90.86%) and the non-melancholic (88.30%) group were composed of five or fewer patients.

The atypical group (n =270) reported a total of 168 unique profiles of symptoms of depressive symptoms while the non-atypical (n =1,173) reported 381 such profiles. The ratio of profiles to patients was about twice as high in the atypical group (0.62) than in the non-atypical group (0.32). In a permutation test, the difference in the number of profiles between the atypical and non-atypical subgroups was smaller than would be expected by chance (*p* = 0.003; the group membership reduces heterogeneity less than would be expected by chance). Equating the groups on sample size (see Fig. 2B) suggested that differences were not clinically meaningful. Most of the DSM-MDE profiles in the atypical (97.02%) and the non-atypical (88.98%) group were composed of five or fewer patients.

### Sensitivity analyses

We re-ran our analyses relaxing the DSM’s hierarchical rule that prohibits the diagnosis of atypical features if a person meets criteria for melancholic features. More individuals met atypical criteria if we relaxed this rule (n =348) but there was no evidence this was associated with lower heterogeneity: this “atypical” group (n =348) reported a total of 253 unique profiles of symptoms of depressive symptoms plus the atypical specifier with 217 profiles of depressive symptoms alone. The non-atypical group (n = 2,148) reported 1,128 profiles of atypical and depressive symptoms and 580 profiles of DSM-MDE symptoms. The ratio of DSM-MDE plus atypical profiles to patients in the atypical (0.64) and non-atypical (0.52) were closer without the DSM’s hierarchical rule. Similarly, the ratio of DSM-MDE profiles was higher in the atypical (0.62) than the non-atypical group (0.27). As before, both of these differences between the groups were smaller than would be expected by chance (*p*s <0.001).

Additionally, we explored whether any specific symptom, as opposed to specifier groups, were associated with reduced heterogeneity; see Table 2. The results of these analyses suggest that the number of unique symptom combination is associated to the group size such that more infrequently-endorsed symptoms appear less heterogeneous by virtue of having fewer individuals in the subgroup but no symptom appeared to reduce heterogeneity considerably (e.g., most of the ratios shows as many symptom profiles as there are patients).

**Table 2:** Number and ratio, relative to sample size, of DSM-MDE, melancholic, and atypical symptoms, based on symptom endorsed in STAR*D (N=2496)

Symptoms	n	%	profiles	ratio
Sad mood	2332	93.43	1842	0.79
Lost interest	1982	79.41	1530	0.77
Lost pleasure ^a^	1506	60.34	1154	0.77
Appetite/weight decrease^a^	1127	45.15	880	0.78
Appetite/weight increase^b^	680	27.24	586	0.86
Insomnia	2274	91.11	1791	0.79
Hypersomnia^b^	370	14.82	349	0.94
Psychomotor retardation^a^	231	9.25	219	0.95
Psychomotor agitation^a^	760	30.45	666	0.88
Fatigue	2009	80.49	1549	0.77
Worthlessness/guilt	1912	76.60	1482	0.78
Diminished concentration	2196	87.98	1712	0.78
Suicidality	494	19.79	447	0.91
Unreactive mood	1436	57.53	1096	0.76
Distinct quality of mood	1240	49.68	1018	0.82
Depression worse AM^c^	271	10.86	260	0.96
Early morning awakening	1243	49.80	945	0.76
Mood reactivity	1060	42.47	901	0.85
Leaden paralysis	806	32.29	633	0.79
Rejection sensitivity	1295	51.88	1003	0.78

profiles = number of profiles of unique symptom profiles, ratio = ratio of unique profiles to patients (0 = maximum homogeneity, 1 = maximum heterogeneity, ^a^ also a symptom of the ‘melancholic features’ specifier, ^b^ also a feature of the ‘atypical features’ specifier. ^c^ AM = in the morning

## Discussion

Our objective was to explore whether the DSM specifiers for major depression achieve their intended purpose of creating more empirically homogeneous patient subgroups. We computed both the number of unique “symptoms profiles” that result from adding the specifier criteria to the main symptom criteria for MDE as well as the number of unique symptoms profiles of the MDE symptoms alone. Using the DSM specifiers for atypical and melancholic depression did not identify more homogeneous groups of patients, at least as can be ascertained by quantifying unique profiles of symptoms.

However, the differences in number of symptoms profiles between the melancholic and the non-melancholic groups were not statistically significant and in comparing the atypical and non-atypical group we find the differences between the groups are smaller than would be expected by chance. Moreover, equating the number of patients present in the groups by sub-sampling revealed that the differences in symptom profiles between subgroups of patients who met for the specifier subgroup vs. those that did not were also not clinically significant: across most subgroups there were almost as many profiles as there were patients. Therefore, any apparent differences in heterogeneity between subgroups appears to be driven by variations in sample size. We could find no evidence that specifiers reduce heterogeneity in presenting symptoms.

### Limitations and strengths

Patients were excluded from the STAR*D study if they reported psychotic symptoms or bipolar disorder as well as if they were deemed to have primary OCD, substance dependence, and prior non-response to the Level 1 medication (i.e., citalopram). Thus, our results cannot be generalized to all patients undergoing a major depressive episode (e.g., those with a MDE in the context of bipolar II) nor all patients meeting criteria for a unipolar MDE. While our findings do not automatically generalize to all patients with a MDE, we note that the STAR*D exclusion criteria are relatively representative of criteria in clinical trials [36, 37, 43]. The STAR*D sample is a rather large clinical sample, and patients were recruited with relatively minimal entry criteria from both primary and secondary care, increasing external validity. Finally, our objective, was to explore whether the specifiers for MDE reduce heterogeneity. Thus, our results do not speak to whether there are “true” or valid underlying atypical or melancholic subgroups that could predict metrics of interest (e.g. treatment outcomes or underlying differences in vulnerability to depression).

Moreover, we could only analyze symptoms related to the melancholic and atypical specifiers and did not explore psychotic, mixed, anxious, or other specifiers of depression. We quantified heterogeneity by exploring profiles of self-reported symptoms, not biomarkers or other mechanistically-relevant variables. Finally, We made several decisions that likely downplayed the degree of heterogeneity added by specific symptoms. For example, we treated all forms of insomnia (middle, late, and early) as the same symptom of depression. Similarly, we treated changes in appetite as the same as changes in weight.

### Implications

Our results provide little support for the idea that the DSM major depression specifiers reduce heterogeneity in symptom presentations, above and beyond simply identifying smaller groups of patients. Without correcting for sample size or conducting a formal test of statistical significance, our results may have be taken to suggest that the atypical specifier reduced heterogeneity more than the melancholic specifier because there were fewer symptom profiles in the atypical subgroup. However, the differences in the heterogeneity we observed between the atypical and non-atypical subgroups were actually smaller than would be expected by chance.

Melancholia is often touted as a biological specifier of depression identifying a relatively homogeneous group of patients. Our results do not support this assertion, at least when it is measured by number of unique symptom profiles. Some have argued that the DSM definition of melancholia may not capture the “true” construct of melancholia, in part because it is bound by the DSM definition of MDE (i.e., a patient cannot meet for the melancholic specifier without first meeting criteria for the problematic MDE criteria). If melancholia is to be a useful construct, a central task for research will be to define its necessary features [44]. Parker and colleagues have identified psychomotor disturbances and disproportionate reactions to stressors as hallmarks of the melancholic affliction [6, 45]. Nonetheless, neither one of these are necessary for the DSM specifier, even as the manual notes that psychomotor disturbances are “nearly always present” (p. 151). (Incidentally, our data argue against the ubiquity of psychomotor disturbances in melancholic depression, see Table 1.) Psychomotor retardation may appear to reduce heterogeneity in symptom presentations but only because it is an infrequently endorsed symptom of depression. Accordingly, a rarer symptom (e.g., psychosis) might appear to be more effective in reducing heterogeneity, without actually doing so.

Various lines of evidence converge to undermine the validity of the DSM diagnostic specifiers. First, if one relaxes the DSM hierarchical rule of melancholia over atypical, the specifiers often co-occur suggesting that nothing about the DSM criteria identifies unique groups [46]. Second, existing evidence suggests that the specifiers are not temporally stable [47], suggesting they would not be reliable biomarkers of an individual difference. Third, the specifiers do not appear to have prognostic or predictive value [27, 48], at least in predicting response to antidepressants or cognitive behavioral therapy, again questioning the extent to which they identify subgroups of patients who share a biopsychosocial vulnerability. Finally, various contemporary approaches to the conceptualization of psychopathology undermine the DSM’s categorization of mental disorders, including its categorization of specifiers. Chief among these are the NIMH RDoC criteria as well as the network approach to psychopathology which conceptualizes mental disorders as emerging from the dynamic interactions of symptoms and processes. Our results suggest that the specifiers as currently implemented do not decrease heterogeneity. Future proposals to decrease heterogeneity should be demonstrated empirically and not assumed. In particular, we recommend that researchers who argue that a given classification scheme reduces heterogeneity actually test this assumption, and consider the effect of subgroup sample size on apparent heterogeneity.

## Data Availability

The data that support the findings of this study are openly available in NIMH data archive, https://nda.nih.gov/. The code for the analyses can be found here: https://osf.io/v8sbe/.
